# A bis-calixarene from olefin metathesis

**DOI:** 10.1107/S1600536812022325

**Published:** 2012-05-23

**Authors:** Shimelis T. Hailu, Ray J. Butcher, Paul F. Hudrlik, Anne M. Hudrlik

**Affiliations:** a Department of Chemistry, Howard University, 525 College Street, NW, Washington, DC 2059, USA

## Abstract

A ring-closing olefin metathesis reaction of tetra­kis­(all­yl­oxy)calix[4]arene gave the bis­ calixarene, (15*E*,40*E*,60*E*)-65,74-bis­(prop-2-en-1-yl­oxy)-13,18,38,43,58,63-hexa­oxado­deca­cyclo­[28.26.8.7^20,36^.1^11,45^.1^51,55^.0^5,57^.0^7,12^.0^19,24^.0^26,64^.0^32,37^.0^44,49^.1^68,72^]tetra­hepta­conta-1,3,5(57),7,9,11,15,19(24),20,22,26,28,30(64),32,34,36,40,44(49),45,47,51,53,55(65),60,68,70,72(74)-hepta­cosa­ene, C_74_H_68_O_8_. It is a cage formed from two calix[4]arene units joined by butenyl groups at three of the O atoms on the narrow rim. The fourth O atom on each calixarene unit is joined with an allyl group. Each of the calix[4]arene units has a flattened cone conformation in which the all­yloxy-substituted aryl group and the opposite aryl group are close together and almost parallel [dihedral angle between planes = 1.09 (11)°], and the other two aryl groups are splayed outward [dihedral angle between planes = 79.53 (11)°]. No guest mol­ecule (*e.g*. solvent) was observed within the cage. The alkene C atoms of one of the links between the calixarene moieties are disordered over two orientations with occupancies of 0.533 (9) and 0.467 (9).

## Related literature

For structures of simple flattened cone calix[4]arenes, see: Arduini *et al.* (1996*b* ▶); Drew *et al.* (1997 ▶). For the structure of a bis­ calix[4]arene in a flattened cone conformation, see Arduini *et al.* (1995 ▶). For the use of calixarenes in mol­ecular recognition, see: Gutsche (2008) ▶; Asfari *et al.* (2001 ▶). For the use of the olefin metathesis reaction to produce bridged calixarenes, see: Vougioukalakis & Grubbs (2010 ▶); Yang & Swager (2007 ▶). For background to symmetrical calixarenes, see: Andreetti *et al.* (1983 ▶); Xu *et al.* (1994 ▶). For details of rigidified calixarenes, see: Arduini *et al.* (1996*a* ▶). For their synthesis and characterization, see: Ho *et al.* (1996 ▶); Jaime *et al.* (1991 ▶).
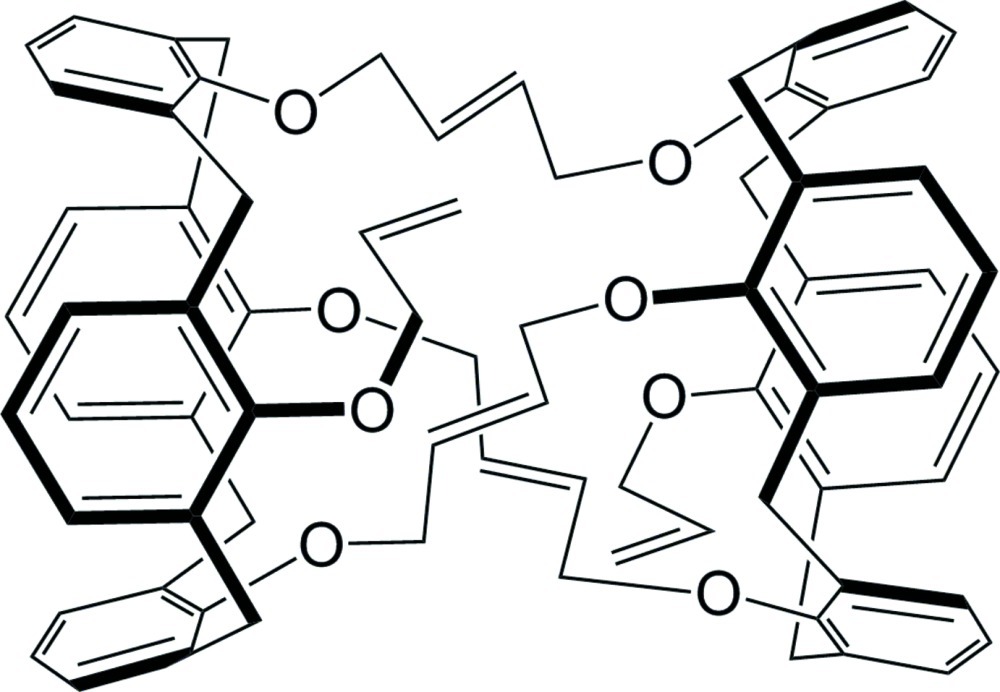


## Experimental

### 

#### Crystal data

C_74_H_68_O_8_
*M* *_r_* = 1085.28
Monoclinic, 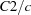

*a* = 29.075 (3) Å
*b* = 12.1376 (11) Å
*c* = 16.9475 (7) Å
β = 94.992 (5)°
*V* = 5958.1 (8) Å^3^
*Z* = 4
Cu *K*α radiation
μ = 0.61 mm^−1^
*T* = 295 K
0.52 × 0.37 × 0.12 mm

#### Data collection

Oxford Diffraction Xcalibur Ruby Gemini diffractometer
Absorption correction: multi-scan (*CrysAlis PRO*; Oxford Diffraction, 2007 ▶) *T*_min_ = 0.836, *T*_max_ = 1.000
10606 measured reflections
5644 independent reflections
3637 reflections with *I* > 2σ(*I*)
*R*_int_ = 0.036

#### Refinement

*R*[*F* ^2^ > 2σ(*F* ^2^)] = 0.072
*wR*(*F* ^2^) = 0.261
*S* = 1.15
5644 reflections
366 parameters
H-atom parameters constrained
Δρ_max_ = 0.30 e Å^−3^
Δρ_min_ = −0.26 e Å^−3^

### 

Data collection: *CrysAlis PRO* (Oxford Diffraction, 2007 ▶); cell refinement: *CrysAlis PRO*; data reduction: *CrysAlis PRO*; program(s) used to solve structure: *SHELXS97* (Sheldrick, 2008 ▶); program(s) used to refine structure: *SHELXL97* (Sheldrick, 2008 ▶); molecular graphics: *SHELXTL* (Sheldrick, 2008 ▶); software used to prepare material for publication: *SHELXTL*.

## Supplementary Material

Crystal structure: contains datablock(s) I, global. DOI: 10.1107/S1600536812022325/hg5194sup1.cif

Structure factors: contains datablock(s) I. DOI: 10.1107/S1600536812022325/hg5194Isup2.hkl

Additional supplementary materials:  crystallographic information; 3D view; checkCIF report

## Figures and Tables

**Fig. 1.**
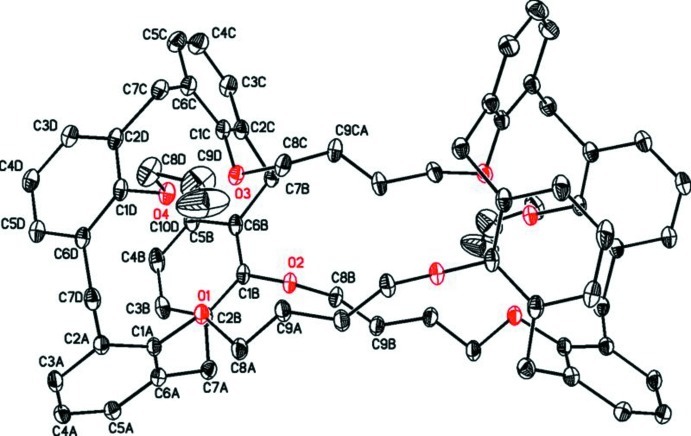
Diagram of C74H68O8 with atomic displacement parameters drawn at 30% probability. Hydrogen atoms are omitted for clarity.

**Fig. 2.**
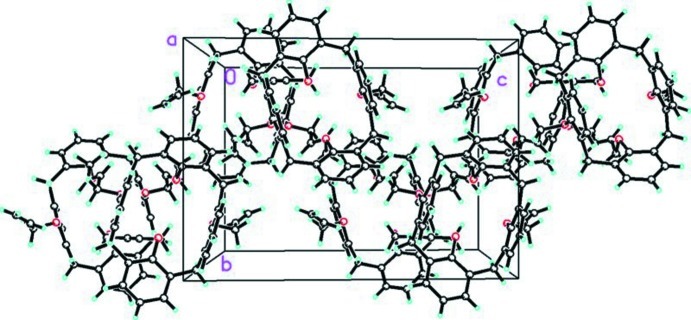
The molecular packing for C74H68O8 viewed along the a axis.

## supplementary crystallographic information

### Comment

Calixarenes are widely used in molecular recognition. They are of particular interest because they can be prepared on large scale and can be modified with a variety of substituents at their upper and lower rims (Gutsche, 2008; Asfari *et al.*, 2001). The olefin metathesis reaction (Vougioukalakis *et al*., 2010) has been used to prepare bridged calixarenes (Yang *et al.*, 2007).

In an attempt to prepare calixarenes with small bridges using ring-closing olefin metathesis, a novel *bis*-calix[4]arene was isolated. The crystal structure shows that the calixarene units in the cage are in a flattened or pinched conformation. For example, the distance across the ring between *para* carbons C4A and C4C is 9.696 (7) Å, while the distance between C4B and C4D is 5.298 (6) Å. The degree of flattening of a cone calix[4]arene is frequently described (Arduini *et al.* 1995; Arduini *et al.* 1996*b*; Drew *et al.* 1997) using the least squares plane of the four bridging methylene groups (C7A, C7B, C7C, C7D) and the dihedral angles of the phenolic rings with this plane. Rings B [90.8 (1)°] and D [91.91 (9)°] are almost perpendicular to this plane, while rings A [136.3 (1)°] and C [144.10 (9)°] are splayed outward. For comparison with more symmetrical calixarenes, equivalent dihedral angles in *t*-butylcalix[4]arene with simple guests are about 123° (Andreetti *et al.*, 1983; Xu *et al.*, 1994) while those in a calix[4]arene rigidified with bridges from diethylene glycol are about 115–118° (Arduini *et al.*, 1996*a*).

### Experimental

A 22-mg (0.027 mmol) sample of first generation Grubbs catalyst was weighed into a 100 ml 3-necked flask in a glove bag under nitrogen. The flask was then connected to a nitrogen line, and 50 ml of dichloromethane (distilled from CaH_2_) followed by 62 mg (0.106 mmol) of tetrakis(allyloxy)calix[4]arene (Ho *et al.*, 1996) in 5 ml of dichloromethane were each added by syringe. The resulting mixture was stirred under reflux (oil bath temperature 45 °C) for 3.5 h. Solvent was removed on a rotary evaporator, and the residue was suspended in 3 ml of dichloromethane and chromatographed (35 g of silica gel, 2.5 *x* 22.5 cm, gradient elution with hexane/dichloromethane). White crystals (2 mg) suitable for X-ray diffraction were obtained from a fraction using hexane/dichloromethane 2:3.

In a similar experiment (10 mg catalyst, 57 ml of dichloromethane, 45 mg of tetrakis(allyloxy)calix[4]arene, 45 °C for 6 h), 22 mg of a white powder was obtained after chromatography, having a nearly identical ^1^H NMR spectrum to the crystals used for X-ray.

The MALDI-TOF MS shows *m/z* 1108.84 ([*M* + Na]^+^ calcd for C_74_H_68_O_8_Na: 1107.48). The ^1^H NMR spectrum includes four doublets between δ 3.0 and 3.3 which show COSY correlations with doublets in the range 4.1–4.7: δ 3.03 (*J* = 13 Hz) and overlapping doublets at 3.20 (*J* = *ca* 14 Hz), 3.23 (*J* = *ca* 13 Hz), and 3.26 (*J* = *ca* 12.5 Hz), correlated with 4.46 (one of two overlapping doublets, *J* = *ca* 13 Hz), 4.38 (*J* = 14 Hz), 4.46 (*J* = *ca* 13 Hz), and 4.59 (*J* = 12.5 Hz), respectively. These are assigned as ArCH_2_Ar protons. (There are additional peaks in the range δ 4.1–4.7 assigned as OCH_2_C=C protons, and a few other COSY correlations within the area.) The HMQC spectrum shows correlations between the ^1^H doublets at δ 3.0–3.3 and ^13^C peaks at δ 30–32, indicating that adjacent phenolic rings in the ArCH_2_Ar are in a *syn* conformation (Jaime *et al.*, 1991), consistent with the cone structure of the calixarene rings. (The ^1^H peaks from 4.1–4.7 correlate with ^13^C peaks at δ 75–76 and at 30–32, confirming that area contains OCH_2_C=C protons as well as ArCH_2_Ar with *syn* phenolic rings.) The remainder of the ^1^H NMR spectrum shows peaks at δ 5.2–5.35 (m, includes 5.30, CH_2_Cl_2_), 5.49 (apparent dq, *J* = 17, 1.5 Hz), 6.15–6.45 (*m*), and 6.7–7.25 [includes 6.87 (t, *J* = 7.5 Hz), 7.02 (apparent t, *J* = *ca* 7.5 Hz), 7.22 (apparent td, *J* = 7.2, 1.6 Hz)]. Other COSY correlations include the peaks in the area about δ 4.2 with δ 5.2–5.35 and the area 6.15–6.45, and the peaks in the areas δ 5.2–5.35 and 5.49 with the area 6.15–6.45.

### Refinement

H atoms were placed in geometrically idealized positions and constrained to ride on their parent atoms with a C—H distances of 0.93 - 0.97Å *U*_iso_(H) = 1.2*U*_eq_(C). The alkene carbon atoms of one of the links between the calixarene moities were disordered over two orientations with occupancies of 0.543 (9) and 0.457 (9). Since this was on a symmetry element the usual idealizing parameters of *SHELXTL* could not be used and its position was generated and then fixed.

### Crystal data

**Table:**

C_74_H_68_O_8_	*F*(000) = 2304
*M**_r_* = 1085.28	*D*_x_ = 1.210 Mg m^−^^3^
Monoclinic, *C*2/*c*	Cu *K*α radiation, λ = 1.54184 Å
Hall symbol: -C 2yc	Cell parameters from 3491 reflections
*a* = 29.075 (3) Å	θ = 4.7–73.7°
*b* = 12.1376 (11) Å	µ = 0.61 mm^−^^1^
*c* = 16.9475 (7) Å	*T* = 295 K
β = 94.992 (5)°	Triangular plate, colorless
*V* = 5958.1 (8) Å^3^	0.52 × 0.37 × 0.12 mm
*Z* = 4	

### Data collection

**Table:**

Oxford Diffraction Xcalibur Ruby Gemini diffractometer	5644 independent reflections
Radiation source: Enhance (Cu) X-ray Source	3637 reflections with *I* > 2σ(*I*)
Graphite monochromator	*R*_int_ = 0.036
Detector resolution: 10.5081 pixels mm^-1^	θ_max_ = 73.8°, θ_min_ = 4.7°
ω scans	*h* = −28→36
Absorption correction: multi-scan (*CrysAlis PRO*; Oxford Diffraction, 2007)	*k* = −13→14
*T*_min_ = 0.836, *T*_max_ = 1.000	*l* = −21→16
10606 measured reflections	

### Refinement

**Table:**

Refinement on *F*^2^	Secondary atom site location: difference Fourier map
Least-squares matrix: full	Hydrogen site location: inferred from neighbouring sites
*R*[*F*^2^ > 2σ(*F*^2^)] = 0.072	H-atom parameters constrained
*wR*(*F*^2^) = 0.261	*w* = 1/[σ^2^(*F*_o_^2^) + (0.1069*P*)^2^ + 5.7734*P*] where *P* = (*F*_o_^2^ + 2*F*_c_^2^)/3
*S* = 1.15	(Δ/σ)_max_ < 0.001
5644 reflections	Δρ_max_ = 0.30 e Å^−^^3^
366 parameters	Δρ_min_ = −0.26 e Å^−^^3^
0 restraints	Extinction correction: *SHELXL97* (Sheldrick, 2008), Fc^*^=kFc[1+0.001xFc^2^λ^3^/sin(2θ)]^-1/4^
Primary atom site location: structure-invariant direct methods	Extinction coefficient: 0.00041 (8)

### Special details

**Table:**

Geometry. All e.s.d.'s (except the e.s.d. in the dihedral angle between two l.s. planes) are estimated using the full covariance matrix. The cell e.s.d.'s are taken into account individually in the estimation of e.s.d.'s in distances, angles and torsion angles; correlations between e.s.d.'s in cell parameters are only used when they are defined by crystal symmetry. An approximate (isotropic) treatment of cell e.s.d.'s is used for estimating e.s.d.'s involving l.s. planes.
Refinement. Refinement of *F*^2^ against ALL reflections. The weighted *R*-factor *wR* and goodness of fit *S* are based on *F*^2^, conventional *R*-factors *R* are based on *F*, with *F* set to zero for negative *F*^2^. The threshold expression of *F*^2^ > σ(*F*^2^) is used only for calculating *R*-factors(gt) *etc*. and is not relevant to the choice of reflections for refinement. *R*-factors based on *F*^2^ are statistically about twice as large as those based on *F*, and *R*- factors based on ALL data will be even larger.

### Fractional atomic coordinates and isotropic or equivalent isotropic displacement parameters (Å^2^)

**Table:**

	*x*	*y*	*z*	*U*_iso_*/*U*_eq_	Occ. (<1)
O1	0.59359 (7)	0.4051 (2)	0.41115 (13)	0.0690 (7)	
O2	0.58527 (7)	0.3355 (2)	0.22594 (12)	0.0681 (7)	
O3	0.58626 (8)	0.1341 (2)	0.35098 (12)	0.0694 (7)	
O4	0.57024 (7)	0.1938 (2)	0.53045 (13)	0.0717 (7)	
C1A	0.63263 (11)	0.4660 (3)	0.43636 (19)	0.0649 (9)	
C2A	0.64766 (11)	0.4639 (3)	0.51682 (19)	0.0665 (9)	
C3A	0.68448 (12)	0.5314 (4)	0.5429 (2)	0.0787 (11)	
H3AA	0.6943	0.5338	0.5965	0.094*	
C4A	0.70666 (13)	0.5947 (4)	0.4906 (3)	0.0814 (11)	
H4AA	0.7306	0.6415	0.5091	0.098*	
C5A	0.69337 (13)	0.5888 (4)	0.4107 (2)	0.0807 (11)	
H5AA	0.7095	0.6289	0.3754	0.097*	
C6A	0.65613 (12)	0.5235 (3)	0.3821 (2)	0.0723 (10)	
C7A	0.64367 (14)	0.5090 (4)	0.2938 (2)	0.0811 (11)	
H7AA	0.6104	0.5117	0.2826	0.097*	
H7AB	0.6569	0.5686	0.2651	0.097*	
C8A	0.55283 (13)	0.4721 (4)	0.4108 (3)	0.0912 (13)	
H8AA	0.5517	0.5225	0.3663	0.109*	
H8AB	0.5541	0.5153	0.4590	0.109*	
C9A	0.51112 (12)	0.4038 (4)	0.4050 (2)	0.0797 (11)	
H9AA	0.5108	0.3431	0.4384	0.096*	
C1B	0.63259 (10)	0.3144 (4)	0.23692 (16)	0.0660 (10)	
C2B	0.66176 (12)	0.3996 (4)	0.26629 (18)	0.0727 (10)	
C3B	0.70895 (13)	0.3795 (5)	0.2737 (2)	0.0897 (13)	
H3BA	0.7291	0.4353	0.2919	0.108*	
C4B	0.72650 (13)	0.2785 (5)	0.2546 (3)	0.1056 (17)	
H4BA	0.7583	0.2673	0.2582	0.127*	
C5B	0.69700 (13)	0.1945 (5)	0.2302 (2)	0.0901 (13)	
H5BA	0.7091	0.1257	0.2196	0.108*	
C6B	0.64943 (11)	0.2099 (4)	0.22110 (17)	0.0712 (10)	
C7B	0.61803 (12)	0.1137 (3)	0.19976 (18)	0.0715 (10)	
H7BA	0.6245	0.0852	0.1484	0.086*	
H7BB	0.5862	0.1385	0.1959	0.086*	
C8B	0.56962 (12)	0.3605 (4)	0.1452 (2)	0.0771 (11)	
H8BA	0.5651	0.2928	0.1150	0.092*	
H8BB	0.5926	0.4045	0.1214	0.092*	
C9B	0.52596 (12)	0.4215 (4)	0.1436 (2)	0.0812 (11)	
H9BA	0.5244	0.4779	0.1804	0.097*	
C1C	0.60871 (11)	0.0377 (3)	0.33555 (18)	0.0663 (9)	
C2C	0.62429 (12)	0.0228 (4)	0.26061 (19)	0.0711 (10)	
C3C	0.64677 (15)	−0.0752 (4)	0.2457 (2)	0.0891 (13)	
H3CA	0.6572	−0.0875	0.1961	0.107*	
C4C	0.65359 (17)	−0.1532 (5)	0.3031 (3)	0.1015 (15)	
H4CA	0.6678	−0.2191	0.2915	0.122*	
C5C	0.63993 (16)	−0.1371 (4)	0.3784 (3)	0.0922 (13)	
H5CA	0.6457	−0.1908	0.4172	0.111*	
C6C	0.61758 (12)	−0.0402 (3)	0.3957 (2)	0.0720 (10)	
C7C	0.60590 (13)	−0.0143 (4)	0.47897 (19)	0.0747 (10)	
H7CA	0.5737	0.0068	0.4780	0.090*	
H7CB	0.6105	−0.0795	0.5118	0.090*	
C8C	0.53692 (13)	0.1233 (4)	0.3494 (2)	0.0798 (11)	
H8CA	0.5299	0.0553	0.3759	0.096*	
H8CB	0.5252	0.1836	0.3795	0.096*	
C9CA	0.5171 (3)	0.0887 (9)	0.2713 (5)	0.074 (2)	0.467 (9)
H9CA	0.5243	0.0164	0.2529	0.089*	0.467 (9)
C9CB	0.50963 (7)	0.1508 (2)	0.27494 (10)	0.074 (2)	0.53
H9CB	0.5119	0.2256	0.2574	0.089*	0.533 (9)
C1D	0.61777 (7)	0.1797 (2)	0.53531 (10)	0.0647 (9)	
C2D	0.63587 (7)	0.0782 (2)	0.51416 (10)	0.0679 (9)	
C3D	0.68347 (7)	0.0648 (2)	0.52266 (10)	0.0782 (11)	
H3DA	0.6962	−0.0024	0.5096	0.094*	
C4D	0.71216 (12)	0.1491 (4)	0.5500 (2)	0.0835 (12)	
H4DA	0.7439	0.1379	0.5573	0.100*	
C5D	0.69375 (12)	0.2501 (4)	0.5666 (2)	0.0763 (11)	
H5DA	0.7133	0.3077	0.5834	0.092*	
C6D	0.64619 (11)	0.2673 (3)	0.55867 (16)	0.0643 (9)	
C7D	0.62721 (12)	0.3825 (3)	0.57134 (19)	0.0730 (10)	
H7DA	0.5939	0.3819	0.5610	0.088*	
H7DB	0.6347	0.4047	0.6259	0.088*	
C8D	0.55093 (15)	0.1671 (6)	0.6025 (3)	0.1125 (18)	
H8DA	0.5545	0.0888	0.6128	0.135*	
H8DB	0.5675	0.2065	0.6460	0.135*	
C9D	0.5037 (3)	0.1951 (8)	0.5987 (6)	0.182 (4)	
H9DA	0.4884	0.1743	0.5506	0.219*	
C10D	0.4787 (4)	0.2373 (8)	0.6397 (10)	0.288 (8)	
H10D	0.4898	0.2620	0.6897	0.345*	
H10E	0.4477	0.2462	0.6223	0.345*	

### Atomic displacement parameters (Å^2^)

**Table:**

	*U*^11^	*U*^22^	*U*^33^	*U*^12^	*U*^13^	*U*^23^
O1	0.0565 (12)	0.0839 (18)	0.0637 (12)	0.0043 (11)	−0.0118 (9)	−0.0064 (11)
O2	0.0485 (11)	0.1003 (19)	0.0542 (11)	0.0027 (11)	−0.0044 (8)	0.0065 (11)
O3	0.0688 (13)	0.0835 (17)	0.0540 (11)	0.0196 (12)	−0.0049 (9)	−0.0080 (11)
O4	0.0535 (12)	0.100 (2)	0.0609 (12)	0.0085 (12)	0.0013 (9)	0.0023 (12)
C1A	0.0536 (16)	0.075 (2)	0.0636 (17)	0.0054 (16)	−0.0106 (13)	−0.0083 (16)
C2A	0.0591 (17)	0.078 (2)	0.0608 (17)	0.0092 (17)	−0.0048 (13)	−0.0108 (16)
C3A	0.067 (2)	0.098 (3)	0.068 (2)	0.007 (2)	−0.0155 (16)	−0.018 (2)
C4A	0.066 (2)	0.080 (3)	0.095 (3)	−0.0015 (19)	−0.0138 (19)	−0.014 (2)
C5A	0.072 (2)	0.078 (3)	0.089 (2)	−0.008 (2)	−0.0087 (18)	0.004 (2)
C6A	0.067 (2)	0.078 (3)	0.069 (2)	0.0019 (18)	−0.0106 (15)	0.0029 (18)
C7A	0.085 (2)	0.088 (3)	0.067 (2)	−0.005 (2)	−0.0115 (17)	0.018 (2)
C8A	0.061 (2)	0.099 (3)	0.108 (3)	0.011 (2)	−0.023 (2)	−0.015 (2)
C9A	0.0617 (19)	0.096 (3)	0.078 (2)	0.0033 (19)	−0.0125 (16)	−0.018 (2)
C1B	0.0465 (15)	0.107 (3)	0.0432 (14)	−0.0035 (17)	−0.0012 (11)	0.0074 (16)
C2B	0.0606 (18)	0.107 (3)	0.0492 (15)	−0.0090 (19)	−0.0032 (13)	0.0087 (17)
C3B	0.059 (2)	0.135 (4)	0.074 (2)	−0.019 (2)	−0.0025 (16)	−0.007 (2)
C4B	0.0482 (19)	0.169 (5)	0.098 (3)	0.003 (3)	0.0008 (18)	−0.024 (3)
C5B	0.0579 (19)	0.132 (4)	0.079 (2)	0.015 (2)	0.0018 (16)	−0.020 (2)
C6B	0.0536 (16)	0.113 (3)	0.0467 (15)	0.0053 (19)	0.0024 (12)	−0.0061 (17)
C7B	0.071 (2)	0.095 (3)	0.0465 (15)	0.0061 (19)	−0.0041 (13)	−0.0080 (16)
C8B	0.0589 (18)	0.108 (3)	0.0619 (18)	0.001 (2)	−0.0093 (14)	0.0184 (19)
C9B	0.062 (2)	0.095 (3)	0.082 (2)	−0.001 (2)	−0.0150 (16)	0.012 (2)
C1C	0.0586 (17)	0.085 (3)	0.0536 (16)	0.0139 (17)	−0.0049 (13)	−0.0087 (16)
C2C	0.0690 (19)	0.089 (3)	0.0535 (16)	0.0106 (19)	−0.0028 (14)	−0.0113 (17)
C3C	0.092 (3)	0.108 (4)	0.067 (2)	0.027 (3)	0.0057 (18)	−0.022 (2)
C4C	0.108 (3)	0.102 (4)	0.095 (3)	0.039 (3)	0.010 (2)	−0.012 (3)
C5C	0.096 (3)	0.094 (3)	0.085 (3)	0.029 (3)	0.000 (2)	−0.001 (2)
C6C	0.070 (2)	0.085 (3)	0.0594 (17)	0.0151 (19)	−0.0034 (14)	−0.0037 (17)
C7C	0.077 (2)	0.087 (3)	0.0585 (18)	0.007 (2)	0.0002 (15)	0.0088 (17)
C8C	0.071 (2)	0.105 (3)	0.0619 (18)	0.029 (2)	−0.0044 (15)	−0.0087 (19)
C9CA	0.054 (3)	0.102 (6)	0.064 (2)	0.018 (4)	−0.0017 (17)	0.007 (4)
C9CB	0.054 (3)	0.102 (6)	0.064 (2)	0.018 (4)	−0.0017 (17)	0.007 (4)
C1D	0.0522 (16)	0.101 (3)	0.0400 (13)	0.0071 (17)	−0.0002 (11)	0.0025 (15)
C2D	0.0634 (18)	0.098 (3)	0.0417 (14)	0.0096 (19)	0.0020 (12)	0.0098 (16)
C3D	0.067 (2)	0.105 (3)	0.0628 (18)	0.018 (2)	0.0035 (15)	0.004 (2)
C4D	0.0539 (18)	0.121 (4)	0.075 (2)	0.015 (2)	0.0009 (15)	0.013 (2)
C5D	0.0571 (18)	0.108 (3)	0.0625 (18)	0.004 (2)	−0.0027 (14)	0.0051 (19)
C6D	0.0594 (17)	0.091 (3)	0.0418 (13)	0.0064 (17)	0.0001 (12)	0.0029 (15)
C7D	0.0664 (19)	0.100 (3)	0.0519 (16)	0.0044 (19)	−0.0002 (14)	−0.0155 (17)
C8D	0.077 (3)	0.166 (5)	0.099 (3)	0.022 (3)	0.031 (2)	0.036 (3)
C9D	0.116 (5)	0.224 (10)	0.218 (8)	0.028 (6)	0.078 (5)	0.044 (7)
C10D	0.197 (10)	0.154 (9)	0.54 (2)	0.044 (8)	0.182 (13)	0.068 (12)

### Geometric parameters (Å, º)

**Table:**

O1—C1A	1.391 (4)	C8B—H8BA	0.9700
O1—C8A	1.437 (4)	C8B—H8BB	0.9700
O2—C1B	1.396 (4)	C9B—C9A^i^	1.316 (5)
O2—C8B	1.437 (4)	C9B—H9BA	0.9300
O3—C1C	1.376 (4)	C1C—C2C	1.397 (5)
O3—C8C	1.439 (4)	C1C—C6C	1.397 (5)
O4—C1D	1.388 (3)	C2C—C3C	1.391 (6)
O4—C8D	1.426 (5)	C3C—C4C	1.360 (7)
C1A—C6A	1.381 (5)	C3C—H3CA	0.9300
C1A—C2A	1.395 (4)	C4C—C5C	1.383 (6)
C2A—C3A	1.390 (5)	C4C—H4CA	0.9300
C2A—C7D	1.509 (5)	C5C—C6C	1.388 (6)
C3A—C4A	1.375 (6)	C5C—H5CA	0.9300
C3A—H3AA	0.9300	C6C—C7C	1.514 (5)
C4A—C5A	1.376 (6)	C7C—C2D	1.511 (5)
C4A—C4C	9.697 (7)	C7C—H7CA	0.9700
C4A—H4AA	0.9300	C7C—H7CB	0.9700
C5A—C6A	1.395 (5)	C8C—C9CA	1.459 (9)
C5A—H5AA	0.9300	C8C—C9CB	1.469 (4)
C6A—C7A	1.519 (5)	C8C—H8CA	0.9701
C7A—C2B	1.516 (6)	C8C—H8CB	0.9699
C7A—H7AA	0.9700	C9CA—H9CA	0.9600
C7A—H7AB	0.9700	C9CB—H9CB	0.9600
C8A—C9A	1.465 (6)	C1D—C6D	1.383 (4)
C8A—H8AA	0.9700	C1D—C2D	1.3991
C8A—H8AB	0.9700	C2D—C3D	1.3884
C9A—C9B^i^	1.316 (5)	C3D—C4D	1.375 (5)
C9A—H9AA	0.9300	C3D—H3DA	0.9300
C1B—C6B	1.394 (5)	C4D—C5D	1.377 (6)
C1B—C2B	1.401 (5)	C4D—H4DA	0.9300
C2B—C3B	1.388 (5)	C5D—C6D	1.393 (5)
C3B—C4B	1.377 (7)	C5D—H5DA	0.9300
C3B—H3BA	0.9300	C6D—C7D	1.526 (5)
C4B—C5B	1.373 (7)	C7D—H7DA	0.9700
C4B—C4D	5.299 (6)	C7D—H7DB	0.9700
C4B—H4BA	0.9300	C8D—C9D	1.411 (8)
C5B—C6B	1.391 (5)	C8D—H8DA	0.9700
C5B—H5BA	0.9300	C8D—H8DB	0.9700
C6B—C7B	1.507 (5)	C9D—C10D	1.166 (12)
C7B—C2C	1.510 (5)	C9D—H9DA	0.9300
C7B—H7BA	0.9700	C10D—H10D	0.9300
C7B—H7BB	0.9700	C10D—H10E	0.9300
C8B—C9B	1.468 (5)		
			
C1A—O1—C8A	110.6 (3)	O3—C1C—C6C	119.8 (3)
C1B—O2—C8B	113.1 (2)	C2C—C1C—C6C	121.4 (3)
C1C—O3—C8C	114.1 (3)	C3C—C2C—C1C	118.1 (4)
C1D—O4—C8D	112.7 (2)	C3C—C2C—C7B	122.1 (3)
C6A—C1A—O1	120.1 (3)	C1C—C2C—C7B	119.8 (3)
C6A—C1A—C2A	122.0 (3)	C4C—C3C—C2C	120.5 (4)
O1—C1A—C2A	117.9 (3)	C4C—C3C—H3CA	119.8
C3A—C2A—C1A	117.8 (4)	C2C—C3C—H3CA	119.8
C3A—C2A—C7D	121.7 (3)	C3C—C4C—C5C	121.8 (4)
C1A—C2A—C7D	120.3 (3)	C3C—C4C—C4A	65.9 (3)
C4A—C3A—C2A	121.1 (3)	C5C—C4C—C4A	67.4 (3)
C4A—C3A—H3AA	119.4	C3C—C4C—H4CA	119.1
C2A—C3A—H3AA	119.4	C5C—C4C—H4CA	119.1
C3A—C4A—C5A	119.8 (4)	C4A—C4C—H4CA	144.5
C3A—C4A—C4C	67.5 (2)	C4C—C5C—C6C	119.4 (4)
C5A—C4A—C4C	66.8 (3)	C4C—C5C—H5CA	120.3
C3A—C4A—H4AA	120.1	C6C—C5C—H5CA	120.3
C5A—C4A—H4AA	120.1	C5C—C6C—C1C	118.7 (3)
C4C—C4A—H4AA	140.7	C5C—C6C—C7C	121.3 (4)
C4A—C5A—C6A	121.0 (4)	C1C—C6C—C7C	119.8 (3)
C4A—C5A—H5AA	119.5	C2D—C7C—C6C	110.7 (3)
C6A—C5A—H5AA	119.5	C2D—C7C—H7CA	109.5
C1A—C6A—C5A	118.0 (3)	C6C—C7C—H7CA	109.5
C1A—C6A—C7A	120.6 (3)	C2D—C7C—H7CB	109.5
C5A—C6A—C7A	121.3 (4)	C6C—C7C—H7CB	109.5
C2B—C7A—C6A	110.1 (3)	H7CA—C7C—H7CB	108.1
C2B—C7A—H7AA	109.6	O3—C8C—C9CA	110.9 (4)
C6A—C7A—H7AA	109.6	O3—C8C—C9CB	117.3 (3)
C2B—C7A—H7AB	109.6	C9CA—C8C—C9CB	31.2 (4)
C6A—C7A—H7AB	109.6	O3—C8C—H8CA	108.5
H7AA—C7A—H7AB	108.1	C9CA—C8C—H8CA	95.3
O1—C8A—C9A	111.0 (4)	C9CB—C8C—H8CA	118.1
O1—C8A—H8AA	109.4	O3—C8C—H8CB	108.4
C9A—C8A—H8AA	109.4	C9CA—C8C—H8CB	124.4
O1—C8A—H8AB	109.4	C9CB—C8C—H8CB	95.3
C9A—C8A—H8AB	109.4	H8CA—C8C—H8CB	107.5
H8AA—C8A—H8AB	108.0	C8C—C9CA—H9CA	118.5
C9B^i^—C9A—C8A	125.3 (5)	C8C—C9CB—H9CB	115.7
C9B^i^—C9A—H9AA	117.3	C6D—C1D—O4	119.3 (2)
C8A—C9A—H9AA	117.3	C6D—C1D—C2D	121.44 (16)
C6B—C1B—O2	120.0 (3)	O4—C1D—C2D	119.20 (15)
C6B—C1B—C2B	121.9 (3)	C3D—C2D—C1D	118.0
O2—C1B—C2B	118.1 (4)	C3D—C2D—C7C	119.31 (17)
C3B—C2B—C1B	117.7 (4)	C1D—C2D—C7C	122.58 (17)
C3B—C2B—C7A	119.5 (4)	C4D—C3D—C2D	121.22 (19)
C1B—C2B—C7A	122.7 (3)	C4D—C3D—H3DA	119.4
C4B—C3B—C2B	121.2 (4)	C2D—C3D—H3DA	119.4
C4B—C3B—H3BA	119.4	C3D—C4D—C5D	119.7 (3)
C2B—C3B—H3BA	119.4	C3D—C4D—C4B	89.82 (19)
C5B—C4B—C3B	119.8 (4)	C5D—C4D—C4B	89.6 (2)
C5B—C4B—C4D	88.0 (3)	C3D—C4D—H4DA	120.1
C3B—C4B—C4D	88.9 (3)	C5D—C4D—H4DA	120.1
C5B—C4B—H4BA	120.1	C4B—C4D—H4DA	90.6
C3B—C4B—H4BA	120.1	C4D—C5D—C6D	120.9 (4)
C4D—C4B—H4BA	93.1	C4D—C5D—H5DA	119.5
C4B—C5B—C6B	121.5 (5)	C6D—C5D—H5DA	119.5
C4B—C5B—H5BA	119.2	C1D—C6D—C5D	118.4 (3)
C6B—C5B—H5BA	119.2	C1D—C6D—C7D	122.0 (3)
C5B—C6B—C1B	117.6 (4)	C5D—C6D—C7D	119.4 (4)
C5B—C6B—C7B	119.9 (4)	C2A—C7D—C6D	110.3 (3)
C1B—C6B—C7B	122.4 (3)	C2A—C7D—H7DA	109.6
C6B—C7B—C2C	111.6 (3)	C6D—C7D—H7DA	109.6
C6B—C7B—H7BA	109.3	C2A—C7D—H7DB	109.6
C2C—C7B—H7BA	109.3	C6D—C7D—H7DB	109.6
C6B—C7B—H7BB	109.3	H7DA—C7D—H7DB	108.1
C2C—C7B—H7BB	109.3	C9D—C8D—O4	111.1 (5)
H7BA—C7B—H7BB	108.0	C9D—C8D—H8DA	109.4
O2—C8B—C9B	109.0 (3)	O4—C8D—H8DA	109.4
O2—C8B—H8BA	109.9	C9D—C8D—H8DB	109.4
C9B—C8B—H8BA	109.9	O4—C8D—H8DB	109.4
O2—C8B—H8BB	109.9	H8DA—C8D—H8DB	108.0
C9B—C8B—H8BB	109.9	C10D—C9D—C8D	137.1 (13)
H8BA—C8B—H8BB	108.3	C10D—C9D—H9DA	111.4
C9A^i^—C9B—C8B	126.1 (4)	C8D—C9D—H9DA	111.4
C9A^i^—C9B—H9BA	116.9	C9D—C10D—H10D	120.0
C8B—C9B—H9BA	116.9	C9D—C10D—H10E	120.0
O3—C1C—C2C	118.7 (3)	H10D—C10D—H10E	120.0
			
C8A—O1—C1A—C6A	−89.4 (4)	C6B—C7B—C2C—C3C	−106.7 (4)
C8A—O1—C1A—C2A	92.6 (4)	C6B—C7B—C2C—C1C	70.9 (4)
C6A—C1A—C2A—C3A	6.9 (5)	C1C—C2C—C3C—C4C	−0.6 (6)
O1—C1A—C2A—C3A	−175.1 (3)	C7B—C2C—C3C—C4C	177.0 (4)
C6A—C1A—C2A—C7D	−167.4 (3)	C2C—C3C—C4C—C5C	−2.0 (8)
O1—C1A—C2A—C7D	10.6 (5)	C2C—C3C—C4C—C4A	−41.5 (3)
C1A—C2A—C3A—C4A	−2.7 (6)	C3A—C4A—C4C—C3C	140.1 (4)
C7D—C2A—C3A—C4A	171.5 (4)	C5A—C4A—C4C—C3C	0.4 (4)
C2A—C3A—C4A—C5A	−2.2 (6)	C3A—C4A—C4C—C5C	−4.1 (3)
C2A—C3A—C4A—C4C	−45.5 (3)	C5A—C4A—C4C—C5C	−143.8 (4)
C3A—C4A—C5A—C6A	3.2 (6)	C3C—C4C—C5C—C6C	1.7 (8)
C4C—C4A—C5A—C6A	46.8 (3)	C4A—C4C—C5C—C6C	40.7 (4)
O1—C1A—C6A—C5A	176.1 (3)	C4C—C5C—C6C—C1C	1.2 (7)
C2A—C1A—C6A—C5A	−6.0 (6)	C4C—C5C—C6C—C7C	−174.3 (4)
O1—C1A—C6A—C7A	−8.1 (5)	O3—C1C—C6C—C5C	179.5 (3)
C2A—C1A—C6A—C7A	169.8 (4)	C2C—C1C—C6C—C5C	−3.9 (6)
C4A—C5A—C6A—C1A	0.8 (6)	O3—C1C—C6C—C7C	−4.9 (5)
C4A—C5A—C6A—C7A	−174.9 (4)	C2C—C1C—C6C—C7C	171.7 (3)
C1A—C6A—C7A—C2B	−75.1 (5)	C5C—C6C—C7C—C2D	109.3 (4)
C5A—C6A—C7A—C2B	100.5 (4)	C1C—C6C—C7C—C2D	−66.2 (4)
C1A—O1—C8A—C9A	−165.0 (3)	C1C—O3—C8C—C9CA	−61.5 (6)
O1—C8A—C9A—C9B^i^	−132.9 (4)	C1C—O3—C8C—C9CB	−95.1 (4)
C8B—O2—C1B—C6B	−82.8 (4)	C8D—O4—C1D—C6D	−90.8 (4)
C8B—O2—C1B—C2B	99.2 (4)	C8D—O4—C1D—C2D	91.1 (4)
C6B—C1B—C2B—C3B	4.8 (5)	C6D—C1D—C2D—C3D	4.56 (16)
O2—C1B—C2B—C3B	−177.2 (3)	O4—C1D—C2D—C3D	−177.40 (13)
C6B—C1B—C2B—C7A	−171.9 (3)	C6D—C1D—C2D—C7C	−172.3 (3)
O2—C1B—C2B—C7A	6.0 (4)	O4—C1D—C2D—C7C	5.71 (19)
C6A—C7A—C2B—C3B	−61.7 (5)	C6C—C7C—C2D—C3D	−60.4 (3)
C6A—C7A—C2B—C1B	115.0 (4)	C6C—C7C—C2D—C1D	116.5 (2)
C1B—C2B—C3B—C4B	−1.5 (6)	C1D—C2D—C3D—C4D	−0.8 (2)
C7A—C2B—C3B—C4B	175.4 (4)	C7C—C2D—C3D—C4D	176.2 (3)
C2B—C3B—C4B—C5B	−2.2 (7)	C2D—C3D—C4D—C5D	−2.5 (4)
C2B—C3B—C4B—C4D	−89.3 (4)	C2D—C3D—C4D—C4B	−91.92 (12)
C3B—C4B—C5B—C6B	2.7 (7)	C5B—C4B—C4D—C3D	1.1 (3)
C4D—C4B—C5B—C6B	90.3 (4)	C3B—C4B—C4D—C3D	121.0 (3)
C4B—C5B—C6B—C1B	0.6 (6)	C5B—C4B—C4D—C5D	−118.6 (4)
C4B—C5B—C6B—C7B	−175.8 (4)	C3B—C4B—C4D—C5D	1.2 (3)
O2—C1B—C6B—C5B	177.7 (3)	C3D—C4D—C5D—C6D	2.2 (5)
C2B—C1B—C6B—C5B	−4.4 (5)	C4B—C4D—C5D—C6D	91.8 (3)
O2—C1B—C6B—C7B	−6.1 (4)	O4—C1D—C6D—C5D	177.1 (2)
C2B—C1B—C6B—C7B	171.8 (3)	C2D—C1D—C6D—C5D	−4.8 (3)
C5B—C6B—C7B—C2C	58.8 (4)	O4—C1D—C6D—C7D	−6.3 (4)
C1B—C6B—C7B—C2C	−117.3 (3)	C2D—C1D—C6D—C7D	171.7 (2)
C1B—O2—C8B—C9B	−158.3 (3)	C4D—C5D—C6D—C1D	1.4 (5)
O2—C8B—C9B—C9A^i^	−135.4 (4)	C4D—C5D—C6D—C7D	−175.3 (3)
C8C—O3—C1C—C2C	102.8 (4)	C3A—C2A—C7D—C6D	−99.0 (4)
C8C—O3—C1C—C6C	−80.5 (4)	C1A—C2A—C7D—C6D	75.0 (4)
O3—C1C—C2C—C3C	−179.7 (3)	C1D—C6D—C7D—C2A	−120.3 (3)
C6C—C1C—C2C—C3C	3.6 (6)	C5D—C6D—C7D—C2A	56.3 (4)
O3—C1C—C2C—C7B	2.5 (5)	C1D—O4—C8D—C9D	172.7 (6)
C6C—C1C—C2C—C7B	−174.1 (3)	O4—C8D—C9D—C10D	−136.3 (13)

Symmetry code: (i) −*x*+1, *y*, −*z*+1/2.

## References

[bb1] Andreetti, G. D., Pochini, A. & Ungaro, R. (1983). *J. Chem. Soc. Perkin Trans. 2*, pp. 1773–1779.

[bb2] Arduini, A., Fanni, S., Manfredi, G., Pochini, A., Ungaro, R., Sicuri, A. R. & Ugozzoli, F. (1995). *J. Org. Chem.* **60**, 1448–1453.

[bb3] Arduini, A., McGregor, W. M., Paganuzzi, D., Pochini, A., Secchi, A., Ugozzoli, F. & Ungaro, R. (1996*a*). *J. Chem. Soc. Perkin Trans. 2*, pp. 839–846.

[bb4] Arduini, A., McGregor, W. M., Pochini, A., Secchi, A., Ugozzoli, F. & Ungaro, R. (1996*b*). *J. Org. Chem.* **61**, 6881–6887. 10.1021/jo960937b 11667582

[bb5] Asfari, Z., Böhmer, V., Harrowfield, J. & Vicens, J. (2001). In *Calixarenes 2001* Dordrecht: Kluwer Academic Publishers.

[bb6] Drew, M. G. B., Beer, P. D. & Ogden, M. I. (1997). *Acta Cryst.* C**53**, 472–474.

[bb7] Gutsche, C. D. (2008). *Calixarenes: An Introduction*, 2nd ed., *Monographs in Supramolecular Chemistry*, edited by J. F. Stoddard. Cambridge: The Royal Society of Chemistry.

[bb8] Ho, Z., Ku, M., Shu, C. & Lin, L. (1996). *Tetrahedron*, **52**, 13189–13200.

[bb9] Jaime, C., de Mendoza, J., Prados, P., Nieto, P. M. & Sánchez, C. (1991). *J. Org. Chem.* **56**, 3372–3376.

[bb10] Oxford Diffraction (2007). *CrysAlis PRO* and *CrysAlis RED* Oxford Diffraction Ltd, Abingdon, England.

[bb11] Sheldrick, G. M. (2008). *Acta Cryst.* A**64**, 112–122. 10.1107/S0108767307043930 18156677

[bb12] Vougioukalakis, G. C. & Grubbs, R. H. (2010). *Chem. Rev.* **110**, 1746–1787. 10.1021/cr9002424 20000700

[bb13] Xu, W., Puddephatt, R. J., Manojlovic-Muir, L., Muir, K. W. & Frampton, C. S. (1994). *J. Inclusion Phenom. Mol. Recognit. Chem.* **19**, 277–290.

[bb14] Yang, Y. & Swager, T. M. (2007). *Macromolecules*, **40**, 7437–7440.

